# Hypofractionated stereotactic radiotherapy for brain metastases in lung cancer patients: dose‒response effect and toxicity

**DOI:** 10.1007/s12672-024-01191-x

**Published:** 2024-07-30

**Authors:** Kaicheng Pan, Bing Wang, Xiao Xu, Jiafeng Liang, Yi Tang, Shenglin Ma, Bing Xia, Lucheng Zhu

**Affiliations:** 1 Department of Radiotherapy, Hangzhou Cancer Hospital, Hangzhou, China; 2 Department of Oncology, Affiliated Hangzhou Cancer Hospital, Zhejiang Chinese Medical University, Hangzhou, China

**Keywords:** Brain metastases, Hypofractionated stereotactic radiotherapy, Lung cancer

## Abstract

**Background:**

Lung cancer is a common cause of brain metastases, approximately 40% of patients with lung cancer will develop brain metastases at some point during their disease. Hypofractionated stereotactic radiotherapy (HSRT) has been demonstrated to be effective in controlling limited brain metastases. However, there is still no conclusive on the optimal segmentation of HSRT. The aim of our study was to explore the correlation between the HSRT dosage and its treatment effect and toxicity.

**Methods:**

A retrospective analysis was conducted on patients with non-small cell lung cancer (NSCLC) brain metastasis at Hangzhou Cancer Hospital from 1 January 2019 to 1 January 2021. The number of brain metastases did not exceed 10 in all patients and the number of fractions of HSRT was 5. The prescription dose ranges from 25 to 40 Gy. The Kaplan–Meier method was used for estimation of the localised intracranial control rate (iLC). Adverse radiation effects (AREs) were evaluated according to CTCAE 5.0. This study was approved by the Institutional Ethics Review Board of the Hangzhou Cancer Hospital (#73/HZCH-2022).

**Results:**

Forty eligible patients with a total of 70 brain metastases were included in this study. The 1-year iLC was 76% and 89% in the prescribed dose ≤ 30 Gy and > 30 Gy group, respectively (*P* < 0.05). For patients treated with HSRT combined with targeted therapy, immunotherapy and chemotherapy, the 1-year iLC was 89%, 100%, and 45%, respectively. No significant associations were observed between the number, maximum diameter, location, and type of pathology of brain metastases. The rate of all-grade AREs was 33%. Two patients who received a total dose of 40 Gy developed grade 3 headache, the rest of the AREs were grade 1–2.

**Conclusions:**

Increasing the prescription dose of HSRT improves treatment effect but may also exacerbate the side effects. Systemic therapy might impact the iLC rate, and individualized treatment regimens need to be developed.

## Background

Lung cancer has long been one of the most common tumours with high mortality rates [1]. With the rapid development of modern cancer treatment, even patients with advanced tumours are surviving for longer periods of time. According to previous studies, about 40% of non-small cell lung cancer (NSCLC) patients will develop brain metastases during the course of the disease [2]. The cumulative occurrence of brain metastases has increased in these long-term survivors. Currently, brain radiotherapy remains a crucial treatment option for brain metastases [3].

However, cognitive decline is frequently observed in patients undergoing whole-brain radiotherapy (WBRT) and only one full dose can be delivered safely and efficaciously [4]. Hypofractionated stereotactic radiotherapy (HSRT) has been demonstrated to be effective in controlling limited brain metastases. Previous studies have shown that HSRT offers durable local control with limited toxicity [5, 6]. The primary benefit of HSRT lies in its ability to deliver a greater biologically effective dose (BED) to vital sites while minimizing radiation exposure to healthy tissues, thereby upholding acceptable levels of adverse radiation effects (AREs).

At present, a prescribed dose of 30 Gy in five or six fractions is commonly used in clinical practice. However, a uniform dose might be insufficient for large tumours. A study by Aoyama et al. showed that the 1-year local control rate (LC) was 59% for lesions > 3 cm^3^ and 96% for lesions ≤ 3 cm^3^ [7]. A study by Marcrom et al. showed that treated with fractionated stereotactic radiotherapy the 1-year LC rate was 95% and 75% in lesions < 3 cm and ≥ 3 cm, respectively [8]. A study by Ishihara et al. showed that an increased dose significantly improved the 1-year LC for PTVs ≥ 4 cm^3^ [9]. Although a few researchers have investigated high doses in patients with brain metastases, the optimal treatment modalities and its safety are still inconclusive in clinical practice.

This study reports the clinical and radiographic results of HSRT for brain metastases focusing on assessing elements that influence response to treatment.

## Materials and methods

### Patient selection

A retrospective analysis was performed in patients with 1–10 brain metastases treated with 5-fraction HSRT at Hangzhou Cancer Hospital from January 1, 2019, to January 1, 2021. Patient characteristics, treatment regimens, and dosimetric factors were retrospectively obtained for analyses. The inclusion criteria were as follows: (1) patients with histologically confirmed NSCLC; (2) patients with 1 to 10 brain metastases detected by magnetic resonance imaging (MRI); and (3) patients aged 18–75 years old. The exclusion criteria were (1) prior surgery for brain metastasis and (2) prior brain radiotherapy. All patients were treated using LINAC-based HSRT and received 25–40 Gy in 5 fractions. None of the patients had received WBRT before or after HSRT. This study was approved by the Institutional Ethics Review Board of the Hangzhou Cancer Hospital (#73/HZCH-2022) and was in accordance with the 1964 Declaration of Helsinki and its later amendments.

### Treatment

All patients were first immobilised using a thermoplastic mask, then a CT scan of the whole brain was performed with a thickness of between 1.25 and 1.5 mm to obtain Planning-CT images. Subsequently, patients were required to undergo a whole-brain MRI scan that must include three-dimensional gadolinium contrast-enhanced T1-weighted sequence. MRI scan layer thickness consistent with CT. The MRI scanning needed to be completed no more than 2 weeks before the start of treatment. The Planning-CT and MRI images were fused using Varian Eclipse 13.5/Revision 1.0 treatment planning software.

Gross tumour volume (GTV) was delineated based on contrast-enhanced T1-weighted magnetic resonance imaging sequences. Clinical target volume (CTV) was defined identically to the GTV. Planning target volume (PTV) was established as a three-dimensional expansion of 3mm around the GTV. All organs at risk (OARs) within 5 cm of the PTV were contoured. HSRT was administered via Varian True Beam linear accelerators, featuring micro-multileaf collimators and utilising a flattening filter-free beam with 6-MV energy and intensity-modulated radiation therapy (IMRT). All HSRT plans are reviewed and approved by both radiotherapy physicists and clinicians.

Systemic therapy was administered concomitantly at the onset of the first brain metastasis radiotherapy. The systemic therapy was decided by the physician under their clinical experience. Before systemic therapy began, all patients underwent genetic testing, including at least EGFR and ALK. Patients in the chemotherapy and targeted therapy groups did not receive combination systemic therapies. The immunotherapy group includes patients receiving either immunotherapy alone or combined immunotherapy with chemotherapy.

### Follow-up and statistics

Following treatment, gadolinium-enhanced MRI scans were conducted 1 month after HSRT and then every 3 months. The local control of all treated lesions commencing from the initial irradiation day, were assessed utilizing the Kaplan‒Meier method. The treatment response of brain metastases was evaluated in accordance with the Response Assessment in Neuro-Oncology Brain Metastases (RANO-BM) criteria [10].

ILC was defined as no more than a 20% increase in the longest diameter of the target lesion on follow-up T1-weighted sequence gadolinium contrast-enhanced MRI. Local failure (LF) was characterised as a 20% rise in the longest diameter of the target lesion, with at least one lesion demonstrating an absolute increase of 5 mm or more. Evaluation of subcentimeter brain metastases response was also conducted following RANO BM guidelines, employing a minimum threshold of 3 mm size increase to define progression. Intracranial progression-free survival (iPFS) was defined as the duration from the initiation of HSRT to the onset of intracranial progressive disease. AREs were evaluated according to the criteria of the CTCAE 5.0.

Subgroups were assessed using the generalized Wilcoxon test for univariate analysis and the Cox proportional hazard model for multivariate analysis. Variables examined for local control encompassed age, sex, metastasis size, histology, number of metastases, and administration of systemic agents. All statistical tests were two-sided, with a significance level set at *P* < 0.05.

## Results

### Patient and lesion characteristics

The characteristics of these patients and tumours are presented in Table 1. Forty lung cancer patients with 70 brain metastases treated with HSRT were reviewed. The median age was 64 years (range 43–87). The median number of target brain metastases was 1 (range 1–4), and 64% of patients had a single brain metastasis. The median prescription dose was 30 Gy. The median follow-up was 10 months. The median maximum diameter and volume of brain metastases were 1.3 cm (range 0.6–4.0 cm) and 2.4 cm^3^ (range 0.2–16.7 cm^3^), respectively. Among the 70 brain metastases, the most common histology was adenocarcinoma (86%). Over half of the patients were on targeted therapy (63%).

**Table 1:** Patient and tumour/treatment characteristics

Characteristics	No./median (range)	%
Patients	40	
Sex		
Male	25	63
Female	15	37
Age	65.5 (43–87)	
Histology		
Adenocarcinoma	32	80
Squamous cell carcinoma	6	15
Adenosquamous	2	5
Number of treated lesions		
1	22	55
2	9	23
3	6	15
4	3	7
Systemic therapy		
Chemotherapy	9	22
Targeted therapy	23	58
Immunotherapy	8	20.0
Gene status		
EGFR or ALK mutation	22	55
Wildtype	18	45
Tumour		
Tumour number	70	100.0
Tumour diameter	1.3 (0.6–4.0) cm	
Tumour volume	2.4 (0.2–16.7) cm^3^	
Histology		
Adenocarcinoma	60	86
Squamous cell carcinoma	7	10
Adenosquamous	3	4
Systemic therapy		
Chemotherapy	15	21
Targeted therapy	44	63
Immunotherapy	11	16
Gene status		
EGFR or ALK mutation	44	63
Wildtype	26	37
Prescription dose		
≤ 30Gy	36	51
> 30Gy	34	49

### Intracranial efficacy

**Fig.1:**
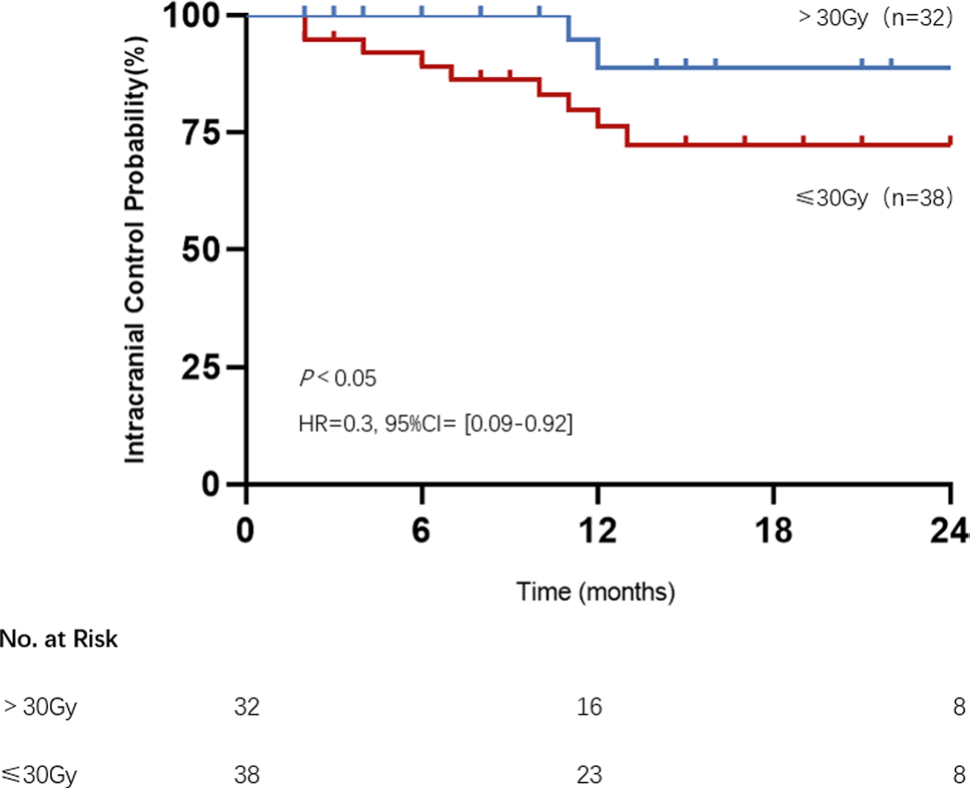
The intracranial control probability of brain metastases according to prescription doses of ≤ 30 Gy vs. > 30 Gy

We recorded the greatest change from baseline in the longest diameter of treated brain metastases and evaluated treatment efficacy in RANO BM criteria. The 1-year iLC rate of all lesions was 80%. For those who developed local failure, the median iPFS was 9 (range 2–13) months. At the cut-off date, intracranial progression occurred in 12 of the 70 lesions. The 1-year iLC was 76% for patients treated with ≤ 30 Gy vs. 89% for patients treated with > 30 Gy (*P* < 0.05, HR = 0.3, 95% CI [0.09–0.92], Fig. 1).

**Fig.2:**
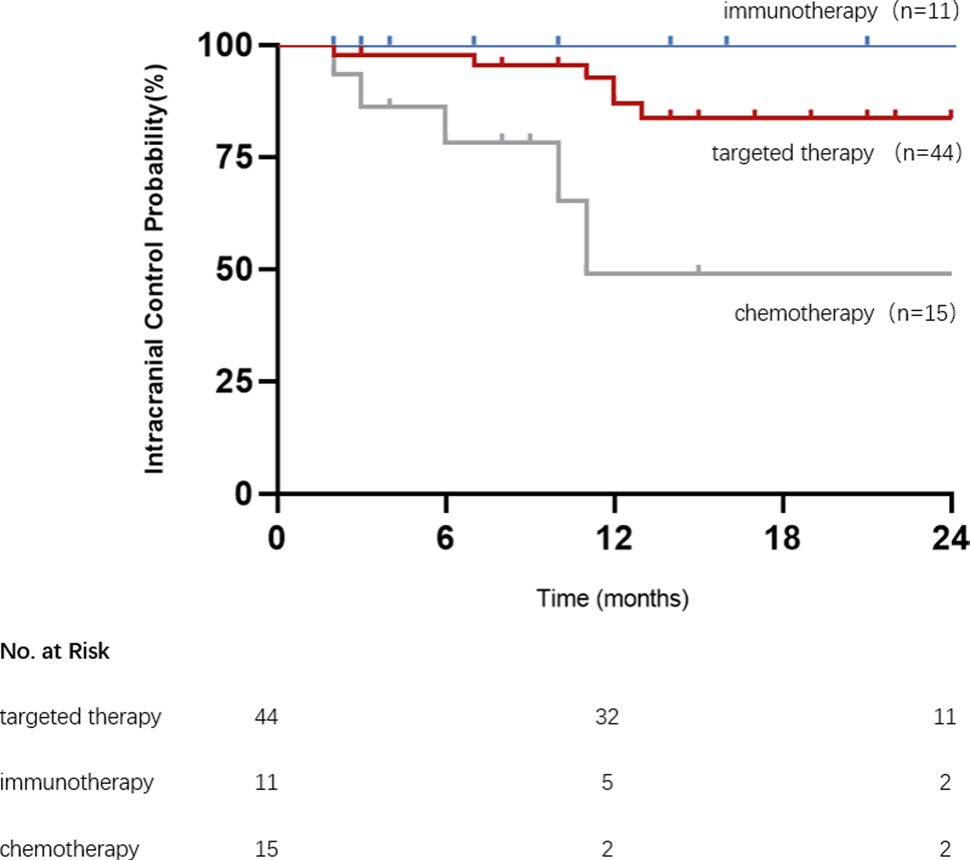
The intracranial control probability of brain metastases according to systemic therapy

Systemic therapy was also associated with the local control rate (Fig. 2). For patients treated with HSRT combined with targeted therapy, immunotherapy and chemotherapy the 1-year iLC was 89%, 100% and 45%, respectively. The 1-year iLC of patients receiving HSRT combined targeted therapy was significantly higher than patients receiving HSRT combined chemotherapy (*P* < 0.01, HR = 0.08, 95% CI [0.01–0.42]).

**Fig.3:**
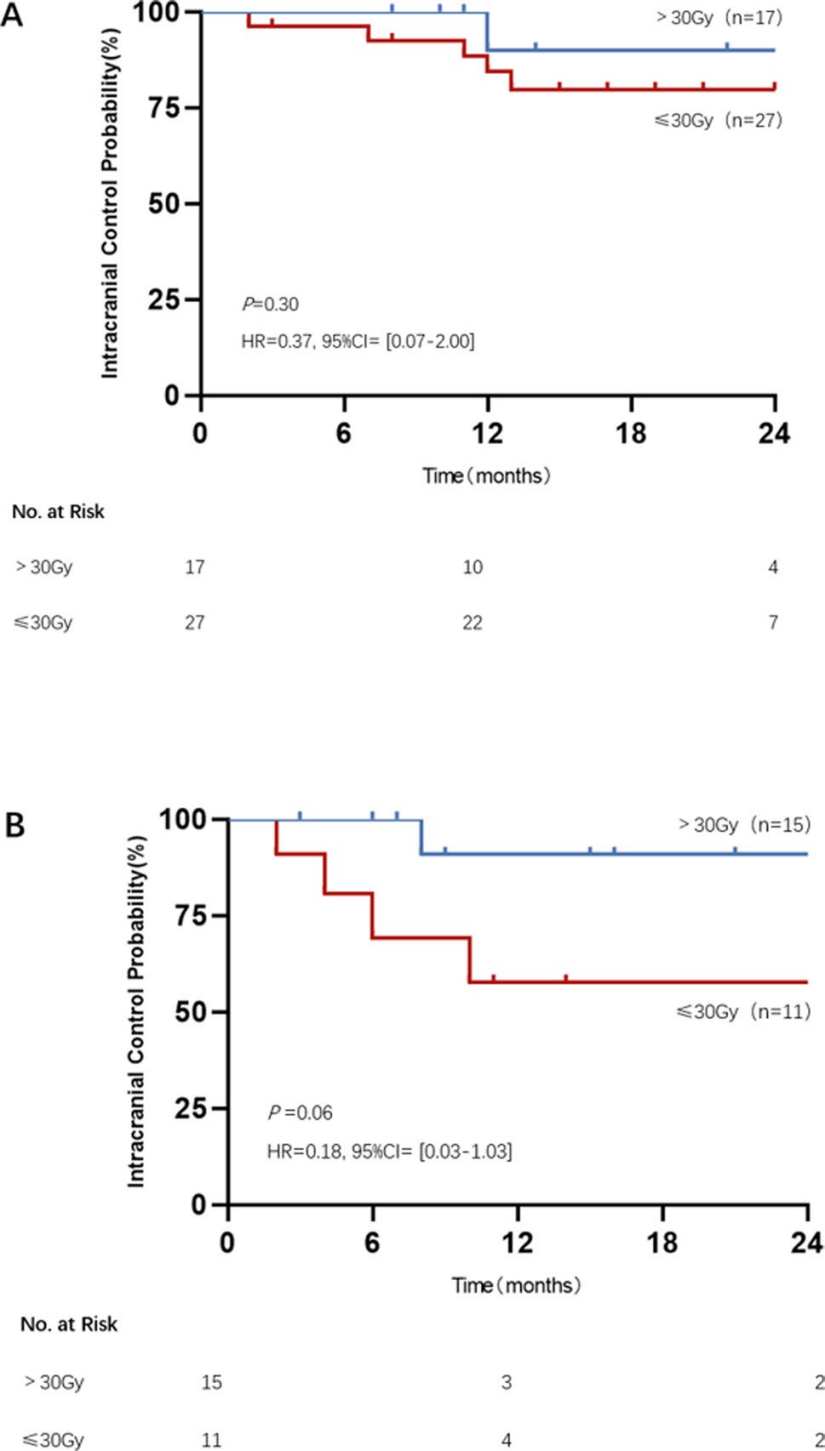
The intracranial control probability of brain metastases according to prescription doses of ≤ 30 Gy vs. > 30 Gy. **A** EGFR or ALK mutation group; **B** without EGFR or ALK mutation group

According to the gene mutation status, we performed further analyses by dividing the lesions into EGFR or ALK mutation group and the no mutation group. In the EGFR or ALK mutation group, the 1-year iLC was 84% for patients treated with ≤ 30 Gy vs. 90% for patients treated with > 30 Gy (*P* = 0.30, HR = 0.37, 95% CI [0.07–2.00], Fig. 3A). The effect of the prescription dose on iLC was larger in those without a EGFR or ALK mutation group, and the 1-year iLC was 58% for patients treated with ≤ 30 Gy vs. 91% for patients treated with > 30 Gy, although this association was not statistically significant. (*P* = 0.06, HR = 0.18, 95% CI [0.03–1.03], Fig. 3B).

Maximal lesion diameter was not found to be a risk factor for 1-year iLC in this model (*P* = 0.39). The 1-year iLC was 84% for large (> 1.5 cm) lesions compared with 80% for small lesions (≤ 1.5 cm).

No statistically significant associations were observed on multivariable regression between tumour location, pathological type, and number of brain metastases with risk of failure.

### Adverse radiation effects

Table 2 presents the incidence of all-grade AREs. The overall rate of AREs was 33%. A total of 13 patients developed an ARE, of which 9/13 occurred within 6 months, with a median time of 5.8 months. The most frequent AREs included headache (7/13), localized edema (2/13), nausea (2/13), hearing loss (1/13), ataxia (1/13), and memory impairment (1/13). 2 patients who received a total dose of 40 Gy developed grade 3 headache, the rest of the AREs were grade 1–2.

**Table 2:** Adverse radiation effects

AREs, No	Prescription dose	Grade (1–2)	Grade (3)	All grades
Headache	≤ 30Gy	3		3
	> 30Gy	2	2	4
Localized edema	≤ 30Gy	1		1
	> 30Gy	1		1
Nausea	≤ 30Gy			
	> 30Gy	2		2
Hearing loss	≤ 30Gy	1		1
	> 30Gy			
Ataxia	≤ 30Gy			
	> 30Gy	1		1
Memory impairment	≤ 30Gy			
	> 30Gy	1		1

## Discussion

Brain metastasis usually has a bleak prognosis. Radiation therapy is a crucial element in the treatment of brain metastases. The challenge in the treatment of brain metastases lies in the development of individualised treatment plans based on the patient's condition. The results of this study indicate that 5-fraction HSRT is effective in treating brain metastases. We report an overall 1-year iLC of 84% for all brain metastasis, and an acceptable ARE rate of 33%. Higher HSRT doses may be correlate with increased iLC, and proper increase of prescription dose will not cause the side effects to increase significantly. These findings could inform clinical practice and the design of future prospective HSRT trials.

HSRT is currently one of the mainstays of brain metastasis treatment. Previously reported 1-year iLC for HSRT have been estimated to be between 52 and 95% [11–15]. The 1-year iLC in our study appears comparable. We excluded patients who had received prior brain radiotherapy or surgery, thus making the iLC of HSRT more realistic.

Currently, 30 Gy/5 fractions is the proposed treatment [16] and is commonly used in clinical practice. However, enhanced LC was observed with the prescribed dose of treatment exceeding 30 Gy/5 fractions, exhibiting the 1-year iLC was 89%. Comparable findings have been documented [8, 17, 18]. Myrehaug’s study showed that patients treated with < 30 Gy/5 fractions showed a 1-year LF rate of 33%, whereas those treated with ≥ 30 Gy/5 fractions had a lower rate at 19%. Notably, a total dose of < 30 Gy compared to ≥ 30 Gy emerged as a significant predictor of LF (HR: 1.68, P = 0.03) [18]. Baliga et al. found that the likelihood of tumour control rose in correlation with the BED_10_, showing a hazard ratio of 0.77 for every 10 Gy_10_ increase. A 1-year LC rate surpassing 70% appeared achievable with a BED_10_ ranging between 40 and 50 Gy. Furthermore, a BED_10_ of 50 to 60 Gy appeared to secure a 1-year LC rate of at least 80% within 12 months [19]. Our findings lead to similar conclusions. Therefore, our results further support the relationship between local tumour control and radiotherapy dose.

Our data did not find that the tumour diameter affected the rate of 1-year LC after HSRT (*P* = 0.39). For small lesions treated with HSRT, the 1-year LC was comparable to that for small lesions treated with single-fraction SRS. As reported in the literature, for small brain metastasis with either single-fraction SRS or HSRT, the 1-year iLC can reach about 80% [18, 20–22], which is similar to 80% of our research. The significant level of LC noted in this study reinforces the increasing utilisation of HSRT, as suggested in existing literature. Small tumour size, as identified in the literature, serves as a predictive factor for enhanced local tumour control. Therefore, HSRT emerges as a viable alternative for managing small brain metastases [23, 24]. Studies have shown that SRS in the treatment of large lesions compared with small lesions can significantly reduce the local control rate of large lesions. However, when we set the tumour diameter to ≥ 1.5 cm, we did not find that the local control rate decreased. Due to the high ratio of hypoxic cells in large brain metastases, HSRT could potentially offer a benefit in terms of local control compared to single-fraction SRS, particularly for larger lesions. The phenomenon of reoxygenation among surviving hypoxic tumour cells during fractionation renders them more susceptible to subsequent irradiation. Harnessing this reoxygenation effect between dose fractions is crucial and should be fully leveraged in the context of HSRT [25].

Generally, higher iLC was observed in patients receiving targeted therapy or immunotherapy combined with HSRT than in those receiving chemotherapy combined with HSRT. Clinical trials have assessed various common chemotherapies, yet they have not shown a significant advantage in brain metastases [26, 27]. For targeted therapy combined with HSRT, a good response has recently been reported in non-small cell lung cancer patients with brain metastases treated with tyrosine kinase inhibitors (TKIs). Many targeted therapies, such as EGFR-TKIs or ALK-TKIs, have good central nervous system (CNS) penetration with an intracranial response rate of more than 70% [28]. HSRT may be able to disrupt the blood–brain barrier (BBB), thereby increasing the CNS penetration of TKIs [29]. Therefore, combined HSRT and TKIs may prove to be useful. A retrospective study showed that the local control rate of patients with NSCLC brain metastases treated with HSRT in combination with TKIs was higher than that of patients treated with HSRT alone [30]. For patients receiving targeted therapy, a lower dose of brain HSRT can also result in a higher local control rate of brain metastases. In patients with negative driver genes, a higher prescription dose may be required to improve intracranial lesion control, although no significant effect was shown in this analysis (Fig. 3).

For immunotherapy combined with HSRT, radiotherapy has been reported to potentially counteract immunosuppression in the tumour microenvironment through various mechanisms [31–33]. HSRT has been shown to enhance the immune response, increasing the susceptibility of tumour cells to T-cell-mediated killing, thereby enhancing local effects [34]. Another potential benefit of HSRT is the increased promotion of the abscopal effect. This phenomenon involves a systemic anti-cancer response triggered by DNA damage induced by radiation. The ideal radiation dose is crucial for activating the cyclic GMP-AMP synthase and stimulator of interferon genes pathway. This pathway promotes targeted responses of cytotoxic T cells in areas of the body not subjected to radiotherapy, a response that can be amplified through the use of immunotherapeutic drugs [35, 36].

No patients in our study developed severe CNS toxicity after receiving HSRT. According to previous reports, radiotherapy-associated CNS toxicity was usually related to the prescribed dose, PTV volume, dose to normal brain tissue and previous radiotherapy history [7, 12, 37]. HSRT provides better protection of peripheral tissues as it promotes more adequately cellular reoxidation and target volume redistribution compared to the single-fraction SRS [38]. Minniti et al. compared the incidence of radionecrosis after single-fraction SRS with multifraction SRS. The results showed that the probability of radionecrosis after treatment was significantly lower for multifraction SRS compared to single-fraction SRS [39]. Due to the limited sample size and difficulty in identifying whether CNS toxicity is caused by brain metastasis or HSRT, we have not found any predictive factors for toxicity.

There are some limitations in this research. Firstly, the data for the study came from a single institution and the study was analysed retrospectively, this may have led to bias in patient selection and confounding factors have not been accounted. Secondly, our sample size was small, which may have meant that our study lacked the power to find statistically significant effects. We found some numerical difference in the results, however, the observed differences did not reach statistical significance.

## Conclusions

HSRT is a reliable treatment option for brain metastases in patients with NSCLC. Currently, commonly used clinical doses of HSRT may not be optimal. Local control rates also improved when prescribed dose escalation were > 30 Gy in 5 fraction. Therefore, it is crucial to administer different treatment regimens according to the individual conditions of patients.

## Data Availability

Data is provided within the manuscript or supplementary information files.

## Abbreviations

HSRT: Hypofractionated stereotactic radiotherapy
iLC: Intracranial local control
NSCLC: Non-small cell lung cancer
SCLC: Small cell lung cancer
WBRT: Whole-brain radiotherapy
SRS: Stereotactic radiosurgery
BED: Biologically effective dose
AREs: Adverse radiation effects
MRI: Magnetic resonance imaging
GTV: Gross tumour volume
CTV: Clinical target volume
PTV: Planning target volume
IMRT: Intensity-modulated radiation therapy
iPFS: Intracranial progression free survival
TKIs: Tyrosine kinase inhibitors
CNS: Central nervous system
LF: Local failure
